# 

**Published:** 1868

**Authors:** 


### SUBSCRIPTION RECEIPTS FOR THE JOURNAL.

Drs. F. M. Hunt, $5; W. A. Childers, $3; J. B. Wright, $4; C. Pinckney, $8; J. H. Phares, $3; W. H. Peebles, $3; J. E. G. Ferrell, $5; F. M. Thomason, 3; Surgeon-General, $4; A. H. S. Hardin, $3; D. W. Hammond, $3; D. D. Westmoreland, 4; A. F. Beckcom, $3; J. M. C. Rice, $4; W. C. Crossley, $2; J. J. A. Smith, 7; Daniel McCall, $5; G. W. McCurry, $1.50; D. L. Phares, $3.

